# Degradation of Acetaldehyde and Its Precursors by *Pelobacter carbinolicus* and *P. acetylenicus*

**DOI:** 10.1371/journal.pone.0115902

**Published:** 2014-12-23

**Authors:** Alexander Schmidt, Marco Frensch, David Schleheck, Bernhard Schink, Nicolai Müller

**Affiliations:** Department of Biology, University of Konstanz, D-78457, Konstanz, Germany; University of Freiburg, Germany

## Abstract

*Pelobacter carbinolicus* and *P. acetylenicus* oxidize ethanol in syntrophic cooperation with methanogens. Cocultures with *Methanospirillum hungatei* served as model systems for the elucidation of syntrophic ethanol oxidation previously done with the lost “*Methanobacillus omelianskii*” coculture. During growth on ethanol, both *Pelobacter* species exhibited NAD^+^-dependent alcohol dehydrogenase activity. Two different acetaldehyde-oxidizing activities were found: a benzyl viologen-reducing enzyme forming acetate, and a NAD^+^-reducing enzyme forming acetyl-CoA. Both species synthesized ATP from acetyl-CoA via acetyl phosphate. Comparative 2D-PAGE of ethanol-grown *P. carbinolicus* revealed enhanced expression of tungsten-dependent acetaldehyde: ferredoxin oxidoreductases and formate dehydrogenase. Tungsten limitation resulted in slower growth and the expression of a molybdenum-dependent isoenzyme. Putative comproportionating hydrogenases and formate dehydrogenase were expressed constitutively and are probably involved in interspecies electron transfer. In ethanol-grown cocultures, the maximum hydrogen partial pressure was about 1,000 Pa (1 mM) while 2 mM formate was produced. The redox potentials of hydrogen and formate released during ethanol oxidation were calculated to be E_H2_ = -358±12 mV and E_HCOOH_ = -366±19 mV, respectively. Hydrogen and formate formation and degradation further proved that both carriers contributed to interspecies electron transfer. The maximum Gibbs free energy that the *Pelobacter* species could exploit during growth on ethanol was −35 to −28 kJ per mol ethanol. Both species could be cultivated axenically on acetaldehyde, yielding energy from its disproportionation to ethanol and acetate. Syntrophic cocultures grown on acetoin revealed a two-phase degradation: first acetoin degradation to acetate and ethanol without involvement of the methanogenic partner, and subsequent syntrophic ethanol oxidation. Protein expression and activity patterns of both *Pelobacter* spp. grown with the named substrates were highly similar suggesting that both share the same steps in ethanol and acetalydehyde metabolism. The early assumption that acetaldehyde is a central intermediate in *Pelobacter* metabolism was now proven biochemically.

## Introduction

The genus *Pelobacter* embraces strictly anaerobic, Gram-negative *delta-proteobacteria* [1]. In contrast to other *Desulfuromonadales*, *Pelobacter* species are fermenting bacteria incapable of anaerobic respiration. Their fermenting capabilities are regarded as a secondary evolutionary event [2] since genes of key enzymes in their fermentative metabolism are likely to originate from lateral gene transfer [3]. The best investigated species within this genus are *Pelobacter carbinolicus* [4] and *Pelobacter acetylenicus* [5]. Both strains originate from different environments: brackish water sediment or freshwater sewage sludge, respectively. Acetoin degradation by *P. carbinolicus* is a well-studied process [6]–[9] and its ability for indirect iron(III) reduction attracted scientific interest [10]–[12]. The genome of *P. carbinolicus* has been sequenced [13]. *P. carbinolicus* is closely related to *P. acetylenicus* [14]. *Pelobacter* species feed only on a narrow substrate range. *P. carbinolicus* and *P. acetylenicus* degrade acetoin, 2,3-butandiol, ethylene glycol (*P. carbinolicus*) or acetylene (*P. acetylenicus* [15], [16]) in pure culture, or ethanol in coculture with a syntrophic partner. The metabolism of all these substrates includes acetaldehyde as central intermediate which was proposed to be the ecological specialisation or niche of these bacteria [1].

The concept of syntrophy describes a particular kind of symbiotic lifestyle implying a mutual dependence of at least two species to perform a substrate conversion. These organisms represent a metabolic entity [17]. In defined cocultures of *Pelobacter* species with methanogens such as *Methanospirillum hungatei* or *Methanobrevibacter arboriphilus*, *Pelobacter* spp. convert ethanol to acetate and hydrogen or formate as electron shuttle [18], [19]. In a second step, the electron shuttle is used to reduce carbon dioxide to methane by a methanogen, thus closing the redox balance of the overall process. The intermediate hydrogen pressure or formate concentration determines the energy yield ratio of both syntrophic partners. Cocultures of *Pelobacter* species are useful model systems to study syntrophic methanogenic ethanol oxidation [20]. Notably, the exceptional *Methanogenium organophilum* is able to perform ethanol conversion to acetate plus methane non-syntrophically in a single organism [21].

The first methanogenic culture growing on ethanol was obtained by V. L. Omeljanskij about 100 years ago [22]. H. A. Barker revisited these experiments by describing *Methanobacillus omelianskii* [23] which was later identified as a syntrophic coculture of two different organisms [24]. However, this culture was lost, and other cocultures such as *P. acetylenicus* or *P. carbinolicus* together with either *Methanospirillum hungatei* or *Methanobrevibacter arboriphilus*, were studied to understand the biochemistry of syntrophic ethanol oxidation. In this context, the genome of *P. carbinolicus* revealed interesting gene loci: a gene cluster for comproportionating hydrogenases (gene loci Pcar_1602-Pcar_1605 and Pcar_1633-Pcar_1936) as described previously for *Thermotoga maritima* [25], and various acetylating or non-acetylating acetaldehyde dehydrogenases. Some of these putative non-acetylating acetaldehyde: ferredoxin oxidoreductases were annotated as either molybdenum- or tungsten-dependent enzymes, and their possible involvement in ethanol metabolism remained unclear.

In the present study, we determined the activities of the cytoplasmic enzymes that are supposed to be involved in ethanol degradation by *P. acetylenicus* and *P. carbinolicus*, following the original metabolic concept of *M. omelianskii* [26], [27] and subsequent studies [28]–[30]. Since initial experiments indicated that growth of *Pelobacter* species/*M. hungatei* cocultures was affected by tungsten and molybdenum availability the dependence of activity and proteome patterns on these trace metals were studied. We inventoried the most abundant soluble enzymes by 2D PAGE and peptide mass-fingerprinting and identified key players in the metabolism by native activity staining. The relative importance of either hydrogen or formate as syntrophic electron shuttles is still a matter of debate. Highly sensitive detection methods allowed to quantify both potential electron carriers in comprehensive cultivation experiments of both *Pelobacter* species grown on acetoin or ethanol. Finally, the simultaneously measured concentration of educts and products of ethanol oxidation helped to elucidate the energetics of ethanol oxidation.

## Results

### Enzyme activities

The specific enzyme activities detected in both *P. acetylenicus* and *P. carbinolicus* after syntrophic growth with ethanol were highly similar (Table 1). Cytoplasmic protein fractions oxidized ethanol with NAD^+^ to acetaldehyde. This alcohol dehydrogenase activity increased at higher pH or if assayed in the (thermodynamically favored) reductive direction. Acetaldehyde was oxidized further to acetyl-CoA by a coenzyme A-dependent acetylating acetaldehyde dehydrogenase activity. Cytoplasmic fractions also exhibited phosphotransacetylase and acetate kinase activity. These enzymes converted acetyl-CoA to equimolar amounts of acetate and ATP via substrate level phosphorylation. In addition to this reaction sequence, a non-acetylating acetaldehyde dehydrogenase (acetaldehyde: benzyl viologen oxidoreductase) activity was detected as well. This enzyme oxidizes acetaldehyde directly to acetate yielding an electron pair of low redox potential able to reduce benzyl viologen (BV) or methyl viologen (MV). NAD^+^ was not a suitable electron acceptor for this reaction.

**Table 1:** Specific enzyme activities (U per mg protein) in *Pelobacter* species. If not mentioned otherwise, all enzyme assays were done with the *P. carbinolicus/M. hungatei* strain M1h coculture. (DCPIP  =  2,6-dichlorophenolindophenol, n. ac.  =  non-acetylating, BV  =  benzyl viologen, MV  =  methyl viologen, *M. arb*.  =  *Methanobrevibacter arboriphilus*, n. d. =  not determined).

Species	*P. acetylenicus*	*P. carbinolicus*	*P. carbinolicus*	*P. carbinolicus*
Culture	Syntrophic	Syntrophic	Pure	Pure
Substrate	Ethanol	Ethanol	Acetaldehyde	Acetoin
Extract	Cell-free extract	Cytoplasmic fraction	Cytoplasmic fraction	Cytoplasmic fraction
Acetoin: DCPIP oxidoreductase	n. d.	0.0027±0	0.006±0.002	1.3±0.28
Alcohol dehydrogenase, NAD^+^ (oxidative) pH 7.5	0.12±0.01	0.019±0	0.033±0.001	0.24±0.04
Alcohol dehydrogenase, NAD^+^ (oxidative) pH 9.0	0.70±0.13	0.26±0	n. d.	2.78±0.54
Alcohol dehydrogenase, NADH (reductive) pH 7.5	1.12±0.06	0.027±0	n. d.	0.60±0.08
Acetaldehyde dehydrogenase, acetylating	0.08±0.01	1.54±0.49	0.008±0.001	4.21±1.0
Phosphotransacetylase	18.84±3.07	37.8±3.8	n. d.	17.0 [7]
Acetate kinase	2.21±0.19	6.4±0.4	n. d.	12.6 [7]
Acetaldehyde dehydrogenase, n. ac., NAD^+^	0	0	n. d.	0
Acetaldehyde dehydrogenase, n. ac., BV	4.86±1.64	3.65±0.6	0.77±0.08	0.25±0.07
Acetaldehyde dehydrogenase, n. ac., MV	2.33±0.278	0.43±0.11	n. d.	n. d.
Formate dehydrogenase, NAD^+^	0.07±0.01	0.96±0.66	n. d.	n. d.
Formate dehydrogenase, BV	4.09±0.43	23.2±1.8	0.083±0.037	n. d.
Formate dehydrogenase, MV	2.14±0	6.2±0.7	n. d.	n. d.
Formate dehydrogenase, NAD^+^, *M. arb.*-Coculture	n. d.	0.0032±0	n. d.	n. d.
Formate dehydrogenase, BV, *M. arb.*-Coculture	n. d.	0.20±0.02	n. d.	n. d.
Formate dehydrogenase, MV, *M. arb.*-Coculture	n. d.	0.08±0.01	n. d.	n. d.
Hydrogenase, NAD^+^	0	0	n. d.	0
Hydrogenase, BV	136±36	75±15	16.0±2.0	0.81±0.07
Hydrogenase, MV	385±109	91±11	n. d.	n. d.

In bacteria with syntrophic lifestyle, hydrogenase or formate dehydrogenase activities are expected to release electron carriers such as H_2_ and/or formate to the syntrophic partners. No hydrogenase and negligible formate dehydrogenase activity with NAD^+^ as electron acceptor was found in either *Pelobacter* species. However, hydrogen or formate reduced benzyl viologen and methyl viologen at high rates. If *P. carbinolicus* was cocultivated with the hydrogen-only scavenging *Methanobrevibacter arboriphilus* [31], [32], formate dehydrogenase activity of *P. carbinolicus* was 100-fold lower than after cocultivation with *M. hungatei*.

In order to identify enzymes that were specifically induced after growth with ethanol, enzymes were also assayed in *P. carbinolicus* cells after axenic growth on acetoin. The pathway of acetoin degradation had been worked out in the past for this bacterium [7], [8]. Activities of alcohol dehydrogenase, acetylating acetaldehyde dehydrogenase and phosphotransacetylase were detected. Additionally, significantly high activities of non-acetylating acetaldehyde dehydrogenase (acetaldehyde: benzyl viologen oxidoreductase) and hydrogenase activity were found although both are not necessary for acetoin degradation. The activity of the initial enzyme of the acetoin pathway, acetoin: DCPIP oxidoreductase [8], was high after growth with acetoin and far lower after growth with ethanol.

For the first time, *P. carbinolicus* was grown axenically on acetaldehyde and enzyme activities were assayed. Acetoin: DCPIP oxidoreductase was still active since the inoculum had been pre-cultivated on acetoin. Alcohol dehydrogenase was also active whereas acetylating acetaldehyde dehydrogenase, the key enzyme of substrate level phosphorylation, exhibited only minor activity. Instead, a non-acetylating acetaldehyde dehydrogenase activity higher than in acetoin-grown cells was detected. The enzyme systems necessary for syntrophic electron transfer, hydrogenase and formate dehydrogenase, were also present after growth with acetaldehyde.

Initial growth experiments (see S1 Fig.) revealed that molybdenum and tungsten are essential trace elements for the *P. acetylenicus*/*M. hungatei* coculture. In the absence of both trace elements, growth was extremely slow. Cocultures grew best if 100 nM tungstate but no molybdenum was added. Cultures supplied with 150 nM molybdate but no tungstate grew slower during the exponential growth phase than tungstate-supplied cultures. In particular non-acetylating acetaldehyde dehydrogenases and formate dehydrogenases are known to depend on tungsten and/or molybdenum-containing cofactors [33]–[36]. Thus, cytoplasmic fractions of *P. carbinolicus* cells grown on ethanol with either 150 nM molybdate or 300 nM tungstate were assayed for these activities (Table 2). Both activities were largely increased after growth in tungstate-rich medium. Since the non-acetylating acetaldehyde dehydrogenase activity in tungstate-free medium was still high, molybdenum might be a suitable substitute for tungsten although the tungsten-dependent enzymes appeared to be more active. On the contrary, the formate dehydrogenase activity was tungstate-induced to a larger extent (100- to 2700-fold), indicating that tungsten is essential for this enzyme and cannot easily be substituted by molybdenum. Noteworthy, the hydrogenase activity tested as a molybdenum- or tungsten-independent reference activity was the same under both growth conditions.

**Table 2:** Specific enzyme activities (U per mg protein) in cytoplasmic fractions of *Pelobacter carbinolicus* cultivated with and without molybdate and tungstate. (n. ac.  =  non-acetylating, BV  =  benzyl viologen, MV  =  methyl viologen).

Trace metal concentration	0 mM W, 150 nM Mo	300 nM W, 0 mM Mo	W-dep. induction
Hydrogenase, BV	91±16	83±11	1.1-fold
Hydrogenase, MV	115±13	60±6	0.5-fold
Formate dehydrogenase, NAD^+^	0.0025±0	0.25±0.13	100-fold
Formate dehydrogenase, BV	0.06±0.01	163±29	2700-fold
Acetaldehyde dehydrogenase, n. ac., BV	0.29±0.02	10.6±2.2	36-fold
Acetaldehyde dehydrogenase, n. ac., MV	0.70±0.13	4.1±1.3	5.8-fold

### Soluble proteome analysis and activity staining

**Figure 1:**
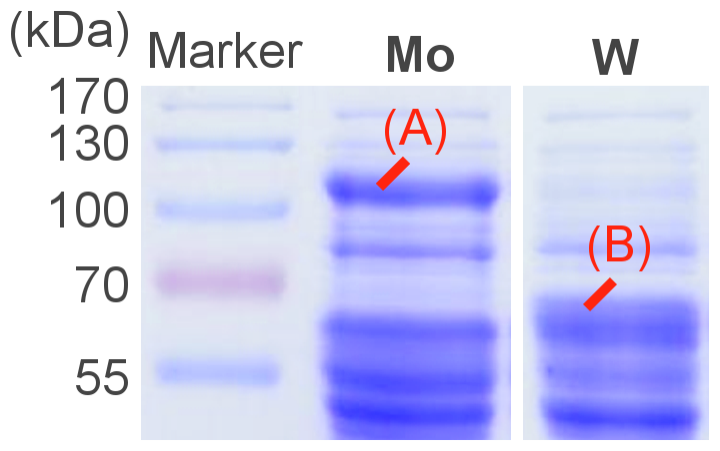
Electrophoretic separation of soluble proteins (20 µg) of *P. carbinolicus* grown in media containing 20 mM ethanol and either 300 nM tungstate without molybdate (W) or 150 nM molybdate without tungstate (Mo). Marked bands were identified as a Mo-dependent acetaldehyde dehydrogenase (A, Pcar_0220) and W-dependent acetaldehyde dehydrogenase isoforms (B, Pcar_0665/0456) by peptide mass fingerprinting.

A possible presence of two types of acetaldehyde dehydrogenases, either molybdenum- or tungsten-dependent, has been discussed in the past [33], [37]. Therefore, we wanted to track down differences in protein expression patterns during growth on ethanol with the named trace element concentrations. As depicted in Fig. 1, simple SDS PAGE of soluble protein extract mainly differed in the expression of two bands which were identified as molybdenum-dependent (Pcar_0220, at 120 kDa, Fig. 1 A) or tungsten-dependent acetaldehyde dehydrogenases (Pcar_0665 or Pcar_0456, at 65 kDa, Fig. 1 B), respectively. Pcar_0665 and Pcar_0456 share 75.4% sequence identity and are likely to carry the same physiological function. Protein expression in standard medium (12 nM W plus 150 nM Mo) was similar to tungstate-rich medium without molybdenum (see S2 Fig.).

**Figure 2:**
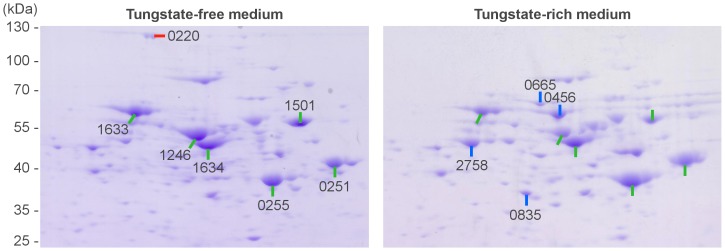
Two-dimensional PAGE comparison of soluble proteins of *P. carbinolicus* grown on 20 mM ethanol in tungstate-free or tungstate-rich medium to identify differentially induced (red or blue) or constitutively (green) expressed proteins by peptide mass fingerprinting. Spots are labeled by locus tag of the identified protein: Pcar_0833/0835  =  formate dehydrogenase, Pcar_1633/1634  =  hydrogenase, Pcar_1501  =  glutamine synthetase, Pcar_1246/2758  =  acetylating acetaldehyde dehydrogenase, Pcar_0251/0255  =  alcohol dehydrogenase isoforms.

A differential analysis of 2D gels in Fig. 2 confirmed the overexpression of both tungsten-dependent isoforms in tungstate-rich media. After growth with only molybdate the large Pcar_0220 was expressed. The spot size was smaller than expected because, according to our experience, this PAGE method discriminates proteins larger than 80 kDa. Besides non-acetylating acetaldehyde dehydrogenase enzymes, 2D PAGE also made putative subunits of a formate dehydrogenase gene cluster (Pcar_0833-0835) detectable which was less abundant. Furthermore, an additional acetylating acetaldehyde dehydogenase (Pcar_2758) was expressed in tungstate-rich medium.

Besides the differentially expressed proteins, 2D PAGE revealed highly abundant and constitutively expressed proteins such as alcohol dehydrogenases (Pcar_0251 and Pcar 0255), an acetylating acetaldehyde dehydrogenase (Pcar_1246), a glutamine synthetase (Pcar_1501, likely responsible for ammonia uptake) and two subunits of a putative hydrogenase (Pcar_1633 and Pcar_1634).

To link activity and protein identification, we applied our recently described activity staining method [38] to cytoplasmic fractions of ethanol-grown *P. carbinolicus* cells (see S3 Fig., Table S1). In all experiments, several spots or lanes were stained per gel strip, indicating that the native protein complexes decayed to varying extent. However, all staining reactions finally led to the same gene locus, confirming the PAGE results and the annotation of the non-acetylating acetaldehyde dehydrogenase (Pcar_0220 with molybdate and Pcar_0665 or Pcar_0456 with tungstate), hydrogenase (Pcar_1633/Pcar_1635 or Pcar_1604/Pcar_1605), and formate dehydrogenase (likely Pcar_0834/Pcar_0835). This links the proteome analyses to the viologen-staining activities determined in the cytoplasmic fraction in Table 1 and 2. Interestingly, hydrogenase and alcohol dehydrogenase gene loci were found repoducibly in the same spots which might indicate that these proteins constitute one complex *in vivo*.

### Formation of hydrogen or formate during syntrophic growth

**Figure 3:**
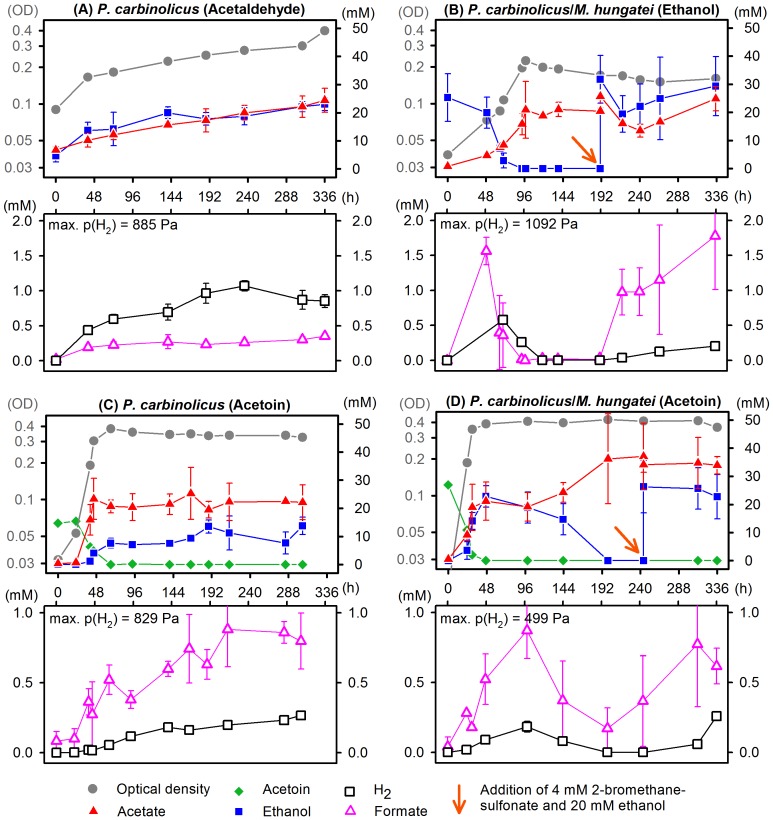
Cultivation of *Pelobacter carbinolicus* either in pure culture or in coculture with *Methanospirillum hungatei* strain M1h on acetaldehyde, ethanol or acetoin. Curves depict concentrations (mM) of acetoin (diamonds), ethanol (squares), acetate (triangles), hydrogen (open squares), formate (open triangles) and the optical density (OD, circles) under different growth conditions. In coculture experiments with ethanol (B) or acetoin (D), the methanogenic partner was inhibited by addition of 4 mM bromoethanesulfonate (BES), and ethanol oxidation was restarted with about 20 mM ethanol (see arrow) after regular growth was finished. Growth and metabolic performance of *P. carbinolicus* was similar to growth of *P. acetylenicus* on acetaldehyde (A) or ethanol (B) as shown in S4 Fig. Concentrations were measured in triplicate.

Growth, substrate turnover and formation of hydrogen or formate by *Pelobacter* species during (syntrophic) growth on acetaldehyde, ethanol or acetoin was evaluated in a 14 days time course (Fig. 3 and S4 Fig.). Moreover, we report about growth of these species on volatile acetaldehyde (Fig. 3 A and Fig. S4). The product formation curves indicate a dissimilation pathway in which acetaldehyde is disproportionated to equimolar amounts of ethanol and acetate.

The data allow a comparison of the formate and the hydrogen pool sizes. As depicted for *P. carbinolicus* in Fig. 3 B, hydrogen partial pressure and formate concentration rose fast during the first hours of syntrophic cultivation with ethanol while only minor amounts of ethanol were consumed. After this burst to about 1,000 Pa or 1 mM hydrogen and 2 mM formate, both concentrations decreased slowly during methanogenic consumption. Concentrations of hydrogen and formate increased again after the methanogen was inhibited by addition of 4 mM bromoethanesulfonate plus 20 mM ethanol. The rate of formation of both electron carriers was exponential and converged a limit. Cultivation of *P. acetylenicus* on ethanol with or without bromoethanesulfonate inhibition showed similar results (see S4 Fig.).

*P. acetylenicus* and *P. carbinolicus* both can grow without a methanogenic partner with acetoin as carbon and energy source, yielding acetate and ethanol as products [4], [5]. Formation of hydrogen during acetoin or ethylene glycol degradation in pure culture has been reported before [39]. Our investigations confirm that in the absence of a methanogenic partner hydrogen and formate were produced in parallel to ethanol, reaching the same maxima (Fig. 3 C). *P. carbinolicus* degraded acetoin in syntrophic coculture as well, producing acetate as the sole product (Fig. 3 D). Growth proceeded in two phases: During a first phase of 48 hours, exponential growth and product formation as in pure culture were observed. In a second phase, the accumulated ethanol was oxidized to acetate as observed with ethanol-degrading cocultures. The course of hydrogen and formate concentrations followed again the ethanol concentration. Bromoethanesulfonate inhibition and ethanol addition caused an increase of hydrogen and formate concentration that came close to the observed limits (Fig. 3 D). The dependence of hydrogen and formate production on the apparent ethanol concentration was observed also in acetaldehyde-grown cultures.

In all cultivation experiments, hydrogen and formate were formed in parallel, even though the turnover of formate was always slightly faster than that of hydrogen. The pool sizes of these electron carriers were within the same order of magnitude. If the headspace was small (330 ml as in ethanol or acetoin cultivations, Fig. 3 B, C, D), the pool size of hydrogen was relatively small. With a bigger headspace (1500 ml as in the acetaldehyde set-up, Fig. 3 A), more electrons were transferred to protons to form hydrogen.

The maximum hydrogen partial pressures and formate concentrations in all cultures allowed the calculation of the corresponding redox potentials using the Nernst equation. The redox potential of hydrogen production was E_H2_ = −358±12 mV, and the redox potential for formate synthesis was E_HCOOH_ = −366±19 mV, assuming a bicarbonate concentration of 60 mM. Thus, both syntrophic electron carriers were released at similar energy levels. The culture-specific redox potentials did not differ between *P. acetylenicus* and *P. carbinolicus* cultures nor between cultivation substrates. Furthermore, simultaneous monitoring of educt and product concentrations in our cultures allowed the calculation of the Gibbs free energy of ethanol oxidation to acetate and hydrogen according to Thauer et al. [40] in ethanol- or acetoin-fed cultures to be ΔG’ = −35 …−28 kJ per mol.

## Discussion

In the present study, key enzymes of ethanol and acetaldehyde metabolism were assayed in two *Pelobacter* species, together with hydrogenase and formate dehydrogenase activities which act as electron carriers to the methanogenic partner organism. The observed similarity of enzyme activities in *P. acetylenicus* and *P. carbinolicus* agrees with the close phylogenetic relationship and other biochemical similarities of these two species [1]. Thus, both species are likely to use the same set of enzymes for ethanol and acetaldehyde metabolism.

### NAD^+^-dependent ethanol oxidation

Both *Pelobacter* species oxidized ethanol with NAD^+^ as electron acceptor, just as the ethanol-oxidizing S organism in the *M. omelianskii* coculture did [26]. However, the standard redox potential of electrons released by ethanol oxidation (−196 mV) is inadequate to reduce NAD^+^ at a standard redox potential of−320 mV, a problem discussed also before [20]. Nonetheless, this unfavorable reaction equilibrium prevents an accumulation of the toxic intermediate acetaldehyde which has been reported to attack DNA and proteins [41]–[43].

### Oxidation of acetaldehyde

Our enzyme assays revealed that acetaldehyde can be oxidized by two different enzymes, an acetylating acetaldehyde dehydrogenase and a non-acetylating acetaldehyde dehydrogenase (acetaldehyde: benzyl viologen oxidoreductase). Acetyl-CoA formation from acetaldehyde and subsequent substrate-level phosphorylation follows the original concept of ethanol oxidation [20], [24], [28], [44] and could easily explain the mode of ATP synthesis and carbon assimilation. Two acetylating acetaldehyde dehydrogenase gene loci could be identified: Pcar_1246 is constitutively expressed and Pcar_2758 was found additionally in fast-growing, tungstate-supplied cultures. Expression and activity of these acetylating acetaldehyde dehydrogenases is in agreement with the detected activities of the subsequent substrate-level phosphorylation enzymes phosphotransacetylase and acetate kinase as determined before [30].

However, the non-acetylating acetaldehyde dehydrogenase activity was constitutively expressed as well. The gene loci Pcar_0456 and Pcar_0665, annotated as tungsten-dependent acetaldehyde: ferredoxin oxidoreductases, were expressed after growth in standard medium (12 nM W plus 150 nM Mo) and were even more expressed in tungstate-rich medium (Fig. 1, Fig. 2 and S2 Fig.). Pcar_0665 and Pcar_0456 share 75.4% sequence identity and are likely to carry the same physiological function. Their annotation as tungsten-dependent enzymes is credible since both enzymes share 34% sequence identity with the well-investigated ferredoxin- and tungsten-dependent acetaldehyde dehydrogenase of *Pyrococcus furiosus* [45]. The well conserved binding motifs of the iron-sulfur clusters, the bound Mg^2+^ and the tungstopterins give further evidence of functional similarities (data not shown). These enzymes were substituted by a molybdenum-dependent isoenzyme (Pcar_0220) if molybdenum but no tungsten was available.

So far, only two mesophilic bacteria were found to be able to express either molybdenum- or tungsten-dependent isoenzymes according to trace metal availability: *Desulfovibrio gigas* [33] and *Eubacterium acidaminophilum* [46]. Other examples of tungsten-dependent acetaldehyde dehydrogenases were found in thermophilic bacteria and archaea [47], [48]. The use of tungsten-dependent isoenzymes in the metabolism of *Pelobacter* species remains unclear. Tungsten cofactors have been reported to catalyse electron transfer reactions at very low redox potential, such as ATP-independent benzoyl-CoA reduction [49]. A tungsten-dependent acetaldehyde dehydrogenase could exploit the entire redox potential of the acetaldehyde/acetate couple (E°' = −580 mV [47]) for ferredoxin reduction and possibly allow for faster acetaldehyde turnover. Coexpression of an additional (tungsten-independent) acetylating acetaldehyde dehydrogenase (Pcar_2758) in *P. carbinolicus* in tungstate-rich medium might help to balance the acetylating and non-acetylating path of acetaldehyde use. Increased substrate turnover can account for the preference of *P. carbinolicus* for tungsten-dependent acetaldehyde dehydrogenases. However, tungstate uptake requires specialised systems and is ATP-consuming in the presence of molybdate since tungstate and molybdate have similar physicochemical properties [50]–[52]. Growth in molybdate-free medium might facilitate tungstate import and with this also expression of tungsten-dependent enzymes. Both arguments could help to explain why *P. acetylenicus* grows faster but not to higher density in tungstate-only medium compared to standard medium (S1 Fig.). The question which one of both acetaldehyde-oxidising pathways is more important for dissimilation of acetaldehyde and its precursors cannot be answered until the acetaldehyde: benzyl viologen oxidoreductase is assayed with its natural electron acceptor. Cell-free extracts of acetaldehyde-grown cells exhibited both activities. In the original concept of acetoin degradation, acetaldehyde was oxidized with NAD^+^, and the formed NADH was used by the NADH-consuming alcohol dehydrogenase to reduce further acetaldehyde to ethanol [6], [7]. However, the non-acetylating acetaldehyde dehydrogenase was also active in cells grown on acetaldehyde or acetoin, indicating an unknown function of this enzyme (Table 1).

### Formation of hydrogen and formate as interspecies electron shuttles

Expression of a comproportionating hydrogenase as proposed by Schut and Adams for the gene loci Pcar_1633-1636 (writing mistake in reference [25] corrected) and Pcar_1602-1605 has been confirmed in the present study. The respective gene loci were the only hydrogenase candidates found in PAGE and activity staining experiments. No NAD^+^-dependent hydrogenase activity was detectable. Hydrogenase activity could be measured with the artificial electron acceptors benzyl viologen and methyl viologen which both can substitute for ferredoxin. Proof of such comproportionation with *in vivo* electron acceptors will be a task for future research. Notably, an NADH- and ferredoxin-dependent hydrogenase was found also in the ethanol-oxidizing S organism of the *M. omelianskii* coculture, which could be interpreted nowadays as a comproportionating hydrogenase activity as well [26].

The measured maximum hydrogen partial pressure (about 1,000 Pa) requires a minimum redox potential of about −358 mV. A similar redox potential could be calculated for formate synthesis (E≈−366 mV). With regard to the detected maximum concentrations, only simultaneous oxidation of NADH and a ferredoxin-like protein (E°'≈−410 mV [53]) would allow for exergonic hydrogenase and formate dehydrogenase reactions. Thus, we expect that the detected tungsten-dependent formate dehydrogenase encoded in gene cluster Pcar_0833-0835 is a comproportionating enzyme as well, even though the formate dehydrogenase exhibited NAD^+^-reducing activity in a reverse assay. All genes encoding formate dehydrogenase subunits show very high sequence identity with the genes of the putative comproportionating hydrogenase gene cluster Pcar_1633-1636 (omitting Pcar_1635 whose function remains unclear). An electron-comproportionating formate dehydrogenase complex has been reported recently for *Clostridium autoethanogenum* [54]. The identified formate dehydrogenase was strictly tungsten-dependent and could not be replaced by molybdenum-dependent isoenzymes, as found in *Desulfovibrio* species [35], [36].

In our experiments, hydrogen could replace formate as syntrophic electron shuttle during ethanol oxidation. Cocultures of *P. carbinolicus* and *Methanobrevibacter arboriphilus* which cannot use formate as electron donor [31], [32] grew at similar rates and to similar densities as cocultures with *M. hungatei* (S5 Fig.). Growth of *Pelobacter carbinolicus* or *Pelobacter acetylenicus* on ethanol solely by interspecies hydrogen transfer in cocultures with *M. arboriphilus*, as well as axenic growth in a culture vessel continuously sparged with nitrogen have also been reported earlier [28], [39], [77]. Enzyme assays revealed that formate dehydrogenase activity in *P. carbinolicus* was lowered by a factor of 100 if formate was not consumed (Table 1). Simultaneous hydrogen and formate turnover (Fig. 3, S4 Fig.) and energetically equivalent maximum concentrations (in connection with synthesis redox potentials, see above) measured in cocultures with *M. hungatei* prove that hydrogen and formate are used equally and probably simultaneously as electron carriers. However, *Pelobacter* species might prefer to use hydrogen as sole electron carrier if tungsten is scarce.

### Syntrophic acetaldehyde oxidation as an ecological niche

The enzyme activity pattern of *P. carbinolicus* cells cultivated with ethanol, acetaldehyde or acetoin (Table 1) revealed a certain degree of substrate adaptation. Nonetheless, few activities could be found which are actually not necessary for the respective metabolism: acetoin: DCPIP oxidoreductase after syntrophic cultivation with ethanol, non-acetylating acetaldehyde dehydrogenase and hydrogenase after axenic cultivation with acetaldehyde or acetoin. A differential 2D PAGE of ethanol- and acetoin-grown cells (data not shown) rendered similar (minor) differences in the expression of these proteins. Obviously, these enzymes are not strictly regulated because the overall spectrum of utilizable substrates of these bacteria is rather limited and comprises only substrates that are all closely related to acetaldehyde. This view is supported also by the intermediate accumulation of side products which are obviously caused also by the comparably high substrate concentrations used.

## Materials and Methods

### Source of organisms

All strains were taken from our own culture collection. *Pelobacter carbinolicus* (strain GraBd1, DSM 2380) and *Pelobacter acetylenicus* (strain WoAcy1, DSM 3246) are available at the German Culture Collection DSMZ. For syntrophic cocultures, *Methanospirillum hungatei* strain M1h (isolated from anoxic sediments of Lake Constance, Germany), *Methanospirillum hungatei* strain JF1 (DSM 846 [55]) and *Methanobrevibacter arboriphilus* (DSM 1125 [31], [32], [56], [57]) were used.

### Cultivation techniques

Cultivation and preparation of anoxic sulfide-reduced, bicarbonate-buffered medium have been described before [58]. *P. acetylenicus* was cultivated in freshwater medium, *P. carbinolicus* in brackish water medium as described [59], with additional 0.25 g/L NH_4_Cl, 1 g/l MgCl_2_·6 H_2_O and without addition of Na_2_SO_4_. Resazurin (0.4 mg/l) was added before autoclaving as redox indicator. Sodium sulfide was used as a 0.5 M stock solution [59]. Vitamins, trace elements and selenite/tungstate were added from 1000-fold concentrated stock solutions. Vitamin solution contained: cyanocobalamin 50, 4-aminobenzoic acid 50, D(+)-biotin 10, nicotinic acid 100, Calcium D(+)-pantothenate 25, pyridoxamine dihydrochloride 250, thiamine dihydrochloride 50 mg/L [59]. EDTA-containing trace element stock solution SL 13 was used [60]. The standard selenite tungstate solution contained 3 mg/L Na_2_SeO_3_·5 H_2_O and 4 mg/L Na_2_WO_4_·2 H_2_O. Molybdate and tungstate concentrations were adjusted by adding or omitting W- or Mo-free stock solutions. Standard medium without modifications contained 12 nM tungstate and 150 nM molybdate.

The medium was prepared in 4 or 10 l flasks and distributed to gas-tight infusion bottles under a headspace of N_2_/CO_2_ (80∶20). Ethanol and acetoin were added from 1 M stock solutions. Cultures were incubated at 28–30°C in the dark. Cell growth was followed microscopically and by measuring the optical density of 1 ml samples at 578 nm against water or dithionite-reduced medium (Hitachi spectrophotometer 100-40).

Growth experiments with *P. acetylenicus* plus *M. arboriphilus* or *M. hungatei* M1h were also done in rubber-stopped Hungate tubes filled with 10 ml brackish water medium that were inoculated with 0.1 ml of both pure cultures grown on acetoin or CO_2_/H_2_ (20%/80%, 1 bar overpressure), respectively. Growth at 30°C was detected as optical density at 600 nm in a tube spectrophotometer (M107, Camspec).

### Growth under trace element limitation

Rubber-stoppered 60 ml-serum bottles that were cleaned by acid and base treatment were extensively flushed with a mixture of N_2_/CO_2_ (80%/20%) to remove oxygen, and autoclaved with 100 µl water. Each bottle was filled by syringes with 30 ml medium prepared without tungstate and molybdate. All trace element limitation experiments were done with medium from the same batch. Each growth condition was checked in triplicate. The specially prepared medium contained 100 nM tungstate or 150 nM molybdate or both trace elements. Precultures for those experiments were grown without tungstate or molybdate once to avoid introduction of tungstate or molybdate to the media by the inoculum. For determination of acetate and ethanol concentrations, 200 µl samples were obtained for HPLC. Methane was measured by gas chromatography at the beginning and the end of the growth experiment.

### Monitoring substrate turnover and hydrogen production

Hydrogen/formate production and substrate turnover in cultures of *Pelobacter* species cultivated on ethanol and acetoin was investigated in triplicate in half-filled infusion bottles with approx. 250 ml medium and 330 ml headspace. Growth experiments with cocultures were done with *M. hungatei* strain M1h as syntrophic partner. Cultures with acetaldehyde as substrate were run semi-sterile in stirred half-filled 1250 ml-infusion bottles with approx. 500 ml medium containing an inoculum of 20% from an acetoin-grown pre-culture. Before inoculation, 500 µl Ti(III)nitrilotriacetic acid (95 mM) was added to eliminate traces of oxygen. Acetaldehyde was added by connecting the headspace of the infusion bottle to a second bottle filled with 2 M anoxic acetaldehyde solution in distilled water via 30 cm gas-tight tubing (5 mm in diameter). The overall headspace was approx. 1500 ml. This set-up was chosen to feed the culture continuously through the gas phase with the volatile acetaldehyde without overdosing this toxic compound. Acetaldehyde cultures were discarded under a fume hood to minimize exposure to ill-smelling thioaldehyde and its derivatives [61], [62]. Hydrogen gas concentrations were measured in triplicate by gas chromatography. These gas concentrations were related to the culture volumes to calculate absolute pool sizes, and related to the liquid culture volume. Formate, acetate, acetoin, and ethanol concentrations were determined in triplicate by HPLC. Methanogenesis was inhibited by addition of 4 mM 2-bromoethanesulfonate.

### Preparation of cell-free extracts of *P. acetylenicus*/*M. hungatei*

Cells grown in 1-l cultures were harvested by centrifugation as described before [60]. Cell pellets were washed twice in anoxic 50 mM potassium phosphate buffer, pH 7.5, containing 3 mM dithiothreitol (DTT). Cell pellets were resuspended in 5 ml of the same buffer, transferred to an 8-ml serum vial sealed with a butyl rubber stopper, shock-frozen in liquid nitrogen, and stored at −20°C. All steps were performed under strictly anoxic conditions in an anoxic glove box [60]. Cell suspensions were lysed by addition of a total of 60 units mutanolysin and 0.2 mg DNaseI per ml cell suspension. Mutanolysin has proven to be a good lysing agent for syntrophic cocultures, as it leaves cell walls of archaea intact and only lyses cells of the fermenting bacteria [63]. Cell suspensions were then incubated anoxically at 37°C for 60 min. Non-lysed cells and debris were removed by centrifugation at 3,000×*g* for 20 min. The supernatant (cell-free extract) was stored in serum vials under N_2_ on ice. Cell-free extracts of *P. acetylenicus* used in enzyme assays were obtained from cocultures with *M. hungatei* strain M1h.

### Preparation of cell-free extracts from *P. carbinolicus*/*M. hungatei* cells

Cell-free extracts of *P. carbinolicus* were prepared using similar techniques as for *P. acetylenicus*. Harvested cell pellets were washed in anoxic 20 mM Tris-HCl (pH 7.3, with medium salts). Pure cultures were lysed directly. In coculture, *P. carbinolicus* cells were separated from the methanogenic partner *M. hungatei* by Percoll gradient centrifugation as described [38]. Solutions of Percoll (Sigma) in harvesting buffer were layered in centrifuge tubes in descending order (concentrations 70%, 65%, 60% and 55%) and the resuspended cell pellet was poured on top. Tubes were centrifuged at 10,000×*g* for 60 min in a swing-bucket rotor. The upper fraction containing *P. carbinolicus* was recovered with a syringe, and purity was checked by light microscopy (purity >99.5%). Cells were washed in harvesting buffer and centrifuged at 16,000×*g* for 15 min.

Separated cells were opened by French Press treatment. 1 µl of each DNase (Fermentas, 1000 U•ml^−1^) and RNase solution (Fermentas, 50,000 U•ml^−1^) and 2 mg of Protease Inhibitor Cocktail P 8465 (Sigma-Aldrich) were added to resuspended *P. carbinolicus* cells. Cells were disrupted anoxically by four passages through a French Pressure Cell (SLM Aminco) at 1,370 bar and ultracentrifuged at 100,000×*g* for 60 min to obtain the cytoplasmic fraction. Non-protein components were removed by gel filtration chromatography on a PD-10 column (GE Healthcare Life Sciences) in an anoxic glove box. Cytoplasmic fractions of extracts of *P. carbinolicus* used in enzyme assays were obtained from cocultures with *M. hungatei* strain M1h.

### Enzyme assays

All enzyme assays were run anoxically in rubber-stoppered 1-ml cuvettes at 30°C in a Hitachi spectrophotometer 100-40 connected to an analogous recorder (BBC Goerz Metrawatt SE 120) as described before [60]. Assays were run in triplicate. One enzyme unit was defined as 1 µmol substrate consumed or product formed per min and mg protein under the respective assay conditions. Benzyl viologen and methyl viologen were regarded as one-electron-acceptors.

Alcohol dehydrogenase (EC 1.1.1.1) was assayed in the oxidative direction in 50 mM Tris/HCl buffer (pH 7.5 or pH 9.0) with 3 mM dithiothreitol (DTT) and 0.25 mM NAD^+^. The reaction was started by addition of 34 mM ethanol. NADH formation was followed at 340 nm (ε = 6.292 mM^−1^•cm^−1^ [64]). The reduction reaction was assayed in 50 mM potassium phosphate buffer (pH 7.5) with 3 mM DTT and 0.2 mM NADH. The reaction was started by addition of 1 mM acetaldehyde and NADH consumption was followed at 340 nm. Non-acetylating acetaldehyde dehydrogenase (EC 1.2.1.3) was assayed as described earlier [33], but with 5 mM acetaldehyde. Activities of phosphotransacetylase (EC 2.3.1.8) was assayed after [65], [66] and acetate kinase (EC 2.7.2.1) by colorimetric determination of acetyl-phosphate consumption after [67] with our own modifications [60]. Non-acetylating acetaldehyde dehydrogenase (EC 1.2.1.3) was assayed in 50 mM potassium phosphate buffer (pH 7.5) with either 0.2 mM NAD^+^ or 2 mM benzyl viologen (BV) or methyl viologen (MV) (modified after [33]). The reaction was started by addition of 5 mM acetaldehyde. NADH formation was followed at 340 nm, BV and MV reduction were followed at 578 nm (ε_BV_ = 8,65 mM^−1^ cm^−1^ [68], ε_MV_ = 9,7 mM^−1^ cm^−1^ [69], [70]). Formate dehydrogenase (EC 1.2.1.2) was assayed in 50 mM potassium phosphate buffer, pH 7.5, 3 mM DTT, 0.3 mM NAD^+^ or 2 mM BV. The reaction was started by addition of 5 mM sodium formate. NADH formation was followed at 340 nm; BV reduction was followed at 578 nm, respectively. Hydrogenase (EC 1.18.99.1) was assayed in 50 mM potassium phosphate buffer, pH 7.5, with 3 mM DTT and 0.25 mM NAD^+^ or 2 mM BV or MV (modified after [70]). Cuvettes were flushed with 100% H_2_ and the potassium phosphate buffer was saturated with H_2_. Cell-free extract was added and the increase of absorption was followed at 578 nm or 340 nm. Acetoin: DCPIP oxidoreductase was assayed in 90 mM imidazole/HCl buffer (pH 7.2) containing 0.08 mM thiamine pyrophosphate, 0.5 mM MgCl_2_, 0.065 mM DCPIP, and 2.5 mM acetoin [6]. DCPIP reduction was followed at 578 nm (ε = 17 mM^−1^ cm^−1^ [6], [71]).

### Protein electrophoresis and activity staining

One-dimensional SDS PAGE was done according to Laemmli [72]. Gel preparation, protein sample preparation, protein electrophoresis and two-dimensional (2D) SDS PAGE were performed according to our previously described protocols [38]. For PAGE analyses, *P. carbinolicus* cocultivated with the genome-sequenced *M. hungatei* strain JF1 were used to exclude possible misidentification of proteins. No contaminants were ever detected. Following observations in initial proteomic experiments, usage of Protease Inhibitor Cocktail P 8465 (Sigma-Aldrich) was necessary. Cell-free extracts from *P. carbinolicus* were desalted by gel filtration with a PD-10 column (GE Healthcare Life Sciences). Isoelectric focusing was extended to a total of 60,000 Vh.

Cytoplasmic extract of *P. carbinolicus* cells cultivated with ethanol was used to identify hydrogenases and formate dehydrogenases in activity staining experiments as described previously [38]. Activities of non-acetylating acetaldehyde dehydrogenases were assayed as for formate dehydrogenase but with 5 mM acetaldehyde as starting reagent. A rather long response time for this enzyme has been observed before [33] which also applied for *P. carbinolicus* activities in cuvette and activity staining test. Hence, the reaction was facilitated by adding 2 µl of freshly prepared, anoxic dithionite solution (about 50 mM).

### Analytical methods

Protein concentrations were measured according to Bradford [73].

Ethanol and acetate concentrations were measured by ion-exclusion HPLC as described before [60], [74]. The HPLC system comprised an autoinjector (Gilson 234; Abimed), a high-pressure pump (LC-10AT vp, Shimadzu), an Aminex HPX-87H column (BioRad) heated to 40°C and a refraction index detector (RID-10A, Shimadzu). 5 mM H_2_SO_4_ was used as eluent at a flow rate of 0.6 ml/min. Acetoin, acetate, and formate were determined using a Shimadzu Prominence HPLC system equipped with a photodiode array detector and an Aminex HPX-87H column (BioRad). 5 mM H_3_PO_4_ was used as eluent at a flow rate of 1 ml/min. The column was heated to 60°C. Acetate and formate were detected at 200±4 nm, acetoin at 274±4 nm. This method was developed to increase the sensitivity of formate detection down to 10 µM. The measured concentrations of acetate were similar for both HPLC systems, which was useful as an internal reference.

Methane was quantified by gas chromatography with a Carlo Erba GC 6000 (Carlo Erba) with a flame-ionization detector by injection of 100 µl sample from the headspace of cultures with gas-tight syringes. Nitrogen was used as carrier gas on a packed CarboSieve column heated to 120°C. Hydrogen was measured by gas chromatography with a Peak Performer 1 RCP (Peak Laboratories) using a reductive gas detector. The two inbuilt columns were heated to 105°C, the detector unit to 265°C. Nitrogen was used as carrier gas. The injected sample size was 100 µl. Besides syringe injection, a gas-tight, self-made pump-through system allowed automatic and highly reproducible injection directly from the culture headspace.

### Peptide mass fingerprinting and database search

Protein bands of interest were excised and analyzed by peptide mass fingerprinting-mass spectrometry at the Proteomics Facility of Konstanz University as described previously [38]. The MASCOT engine (Matrix Science) [75] was used to match each peptide fingerprint against a local database of predicted protein sequences of the annotated genomes of *P. carbinolicus* (NCBI Reference Sequence: NC_007498.2; US DOE Joint Genome Institute; described in [76]) or *M. hungatei* JF1 (NCBI Reference Sequence NC_007796.1; US DOE Joint Genome Institute) to verify cell separation. With the applied procedure, score values of 50 or more are regarded as significant.

### Chemicals

Chemicals were of analytical quality and were obtained from Sigma-Aldrich, Roth or Fluka at. Gases were from Messer Griessheim and Sauerstoffwerke Friedrichshafen at a purity of at least 99,999%.

## Supporting Information

S1 Fig **Trace metal dependent growth of a** ***Pelobacter acetylenicus*** **/** ***Methanospirillum hungatei*** **strain M1h coculture on 20 mM ethanol with 100 nM tungstate and 150 nM molybate (normal medium) (+W +Mo, squares), 100 nM tungstate only (+W -Mo, circles), 150 nM molybate only (-W +Mo, triangles) or without tungstate and molybate (-W -Mo, diamonds).** Depicted are data obtained in triplicate cultures.
(TIF)

S2 Fig **PAGE of soluble proteins of** ***Pelobacter carbinolicus*** **cells grown in coculture with** ***Methanospirillum hungatei*** **on 20 mM ethanol in media of different trace metal composition.** In medium with standard trace metal composition (see material and methods section) and tungsten-supplemented medium (with 300 nM tungstate, W) the tungsten-dependent non-acetylating acetaldehyde dehydrogenases are induced (band at 65 kDa). Under tungsten-limiting condition (with 150 nM molybdate, Mo) the molybdenum-dependent acetaldehyde dehydrogenase is induced (band at 120 kDa).
(TIF)

S3 Fig **Representative activity staining experiments (n = 3) to identify the non-acetylating dehydrogenase, hydrogenase and formate dehydrogenase following our previously described method (Schmidt A, Müller N, Schink B, Schleheck D (2013) A Proteomic View at the Biochemistry of Syntrophic Butyrate Oxidation in Syntrophomonas wolfei.** **PLoS ONE. pp. 1–17**). Soluble extract from *Pelobacter carbinolicus* grown on 20 mM ethanol with different trace element supplements was used. Activity staining was performed anoxically in potassium phosphate buffer (50 mM, pH 7.5) with 2 mM benzyl viologen. Staining was started with 5 mM acetaldehyde, 5 mM formate or pure hydrogen gas, respectively. Addition of dithionite reduced response time of the enzyme reaction. Results of peptide mass fingerprinting are displayed in S1 Table. The unmarked, second spot at the formate dehydrogenase staining developed before formate addition, probably due to hydrogen contamination of the gas phase.
(TIF)

S4 Fig **Growth of representative** ***P. acetylenicus*** **cultures degrading acetaldehyde (A) or ethanol (coculture with** ***M. hungatei*** **JF1; B,D) show same hydrogen or formate production and consumption pattern as the corresponding** ***P. carbinolicus*** **cultures (C, see** **Fig. 3** **in main article).** Inhibition of the methanogen by addition of 2-bromoethanesulfonate (BES, see arrow) and readdition of 20 mM ethanol as substrate (B) led to similiar levels of accumulated hydrogen and formate. (symbols: optical density (filled circles), ethanol (squares), acetate (triangles), hydrogen (open squares) and formate (open triangles)).
(TIF)

S5 Fig **Growth of** ***Pelobacter carbinolicus*** **in coculture with different methanogenic partners on 20 mM ethanol:** ***Methanobrevibacter arboriphilus*** **(blue circles) and** ***Methanospirillum hungatei*** **M1h (red squares).** Depicted are data obtained in triplicate cultures.
(TIF)

S1 Table **Results of peptide mass fingerprinting to identify spots that were stained by non-acetylating acetaldehyde dehydrogenase (acetaldehyde: benzyl viologen oxidoreductase) (AADH), formate dehydrogenase (FDH) and hydrogenase (H_2_ase) activity as shown in** **S3 Fig** **. under different growth conditions.** A score value above 50 is meant to be significant. (Abbreviations: Fd  =  ferredoxin, dep.  =  dependent, su  =  subunit).
(DOCX)
