# Supplementary figures and images for: Degradation of Acetaldehyde and Its Precursors by Pelobacter carbinolicus and P. acetylenicus
Source: PLoS One. 2014 Dec 23;9(12):e115902. doi: 10.1371/journal.pone.0115902 (PMC4275255; doi:10.1371/journal.pone.0115902)

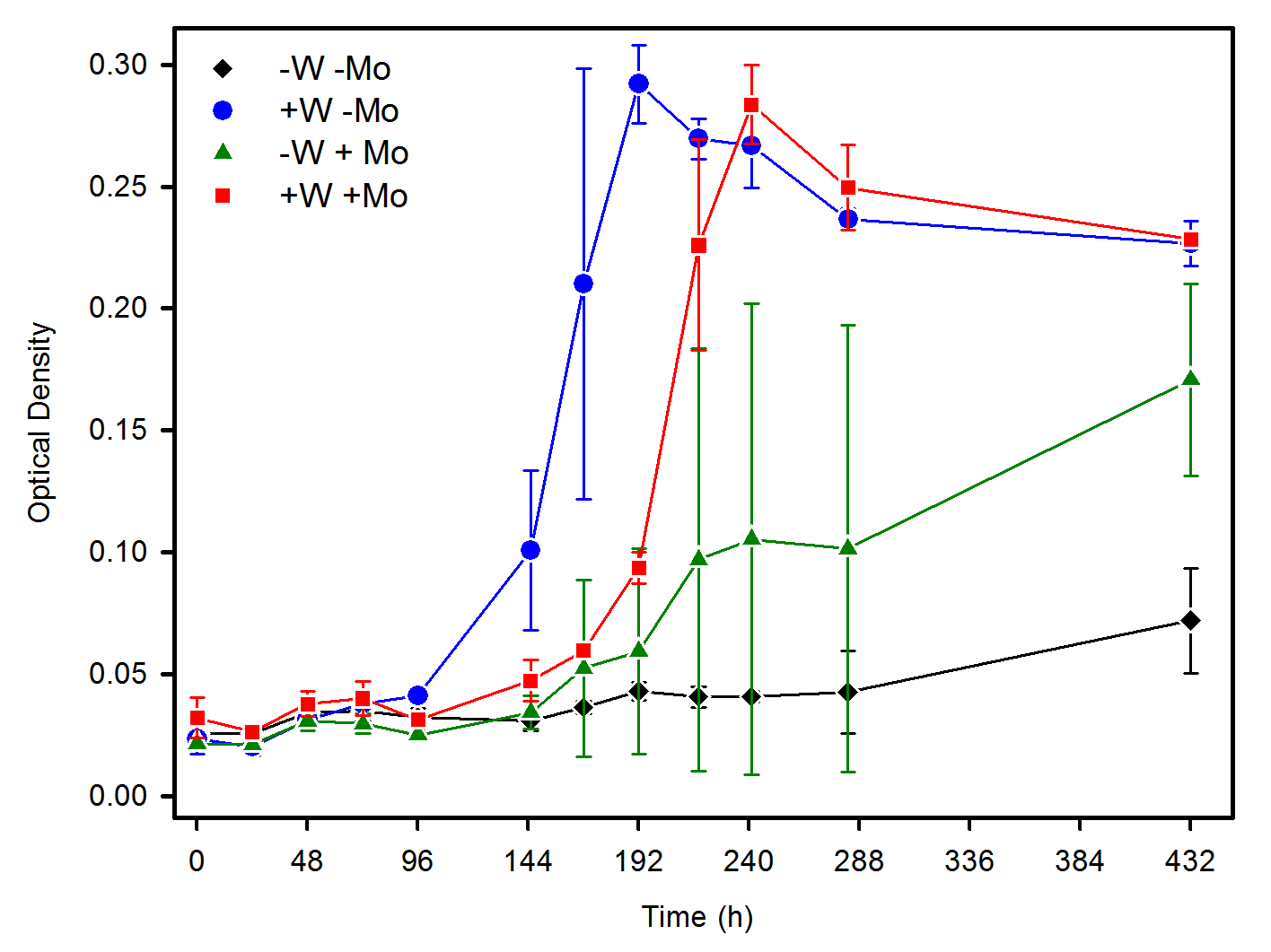

Supplement: S1 Fig — Trace metal dependent growth of a Pelobacter acetylenicus / Methanospirillum hungatei strain M1h coculture on 20 mM ethanol with 100 nM tungstate and 150 nM molybate (normal medium) (+W +Mo, squares), 100 nM tungstate only (+W -Mo, circles), 150 nM molybate only (-W +Mo, triangles) or without tungstate and molybate (-W -Mo, diamonds). Depicted are data obtained in triplicate cultures. (TIF) [file pone.0115902.s001.tif]

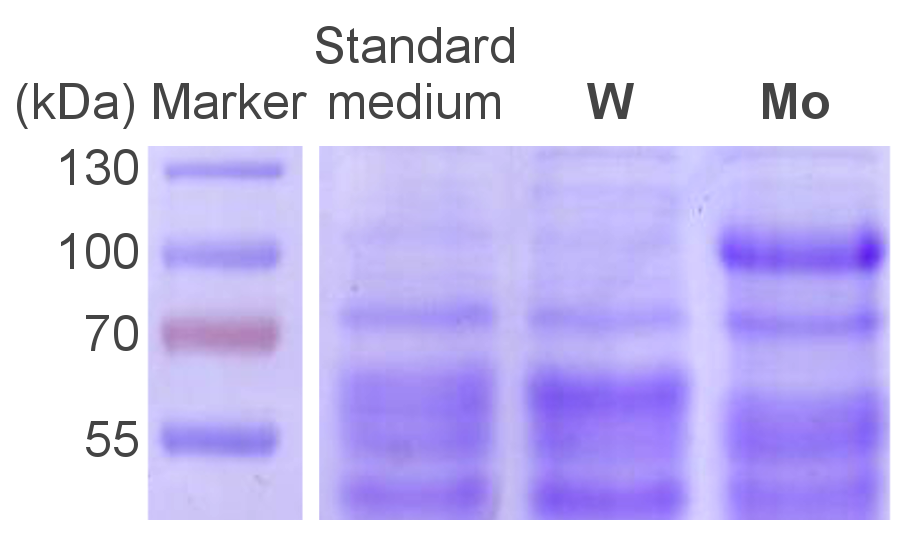

Supplement: S2 Fig — PAGE of soluble proteins of Pelobacter carbinolicus cells grown in coculture with Methanospirillum hungatei on 20 mM ethanol in media of different trace metal composition. In medium with standard trace metal composition (see material and methods section) and tungsten-supplemented medium (with 300 nM tungstate, W) the tungsten-dependent non-acetylating acetaldehyde dehydrogenases are induced (band at 65 kDa). Under tungsten-limiting condition (with 150 nM molybdate, Mo) the molybdenum-dependent acetaldehyde dehydrogenase is induced (band at 120 kDa). (TIF) [file pone.0115902.s002.tif]

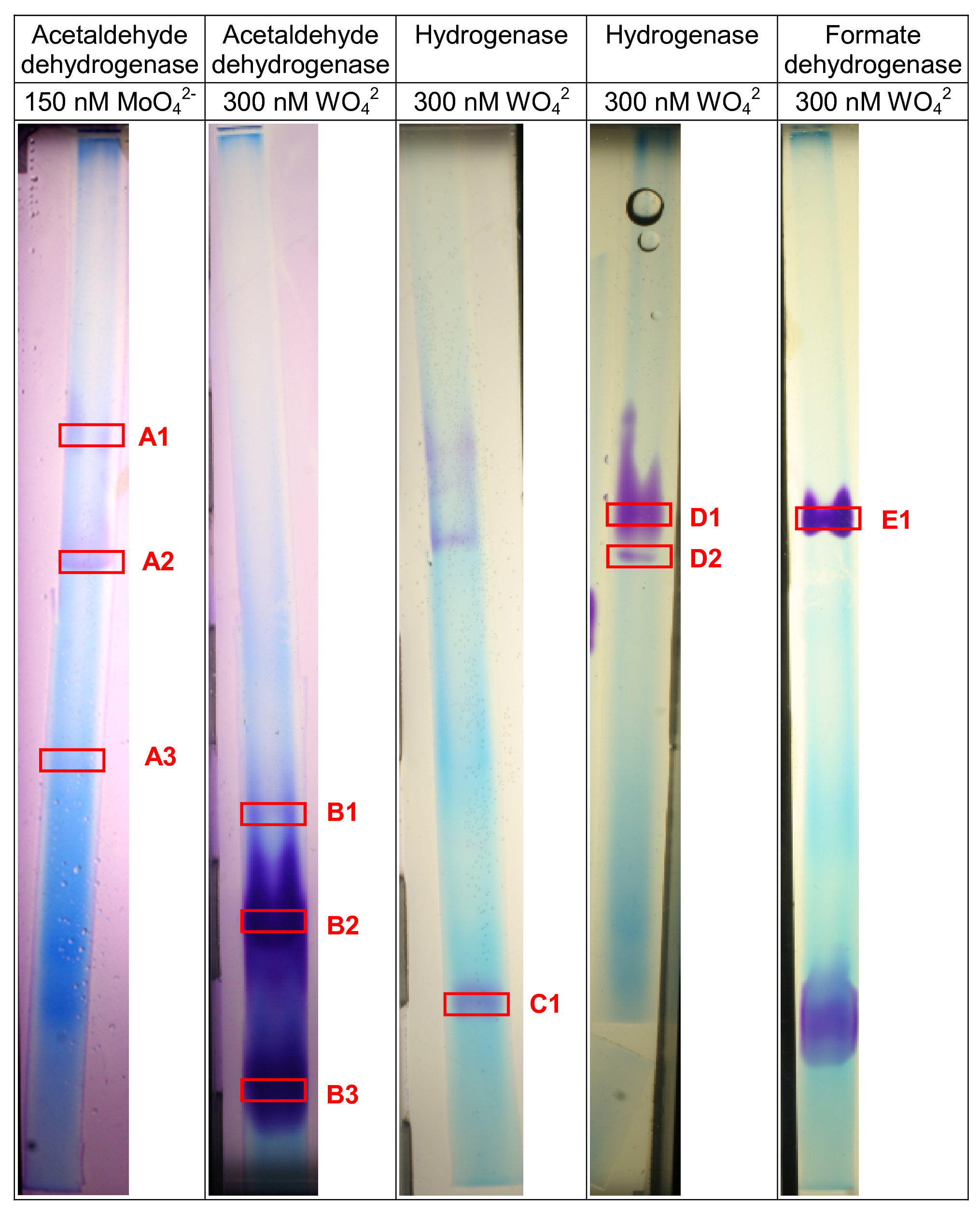

Supplement: S3 Fig — Representative activity staining experiments (n = 3) to identify the non-acetylating dehydrogenase, hydrogenase and formate dehydrogenase following our previously described method (Schmidt A, Müller N, Schink B, Schleheck D (2013) A Proteomic View at the Biochemistry of Syntrophic Butyrate Oxidation in Syntrophomonas wolfei. PLoS ONE. pp. 1–17). Soluble extract from Pelobacter carbinolicus grown on 20 mM ethanol with different trace element supplements was used. Activity staining was performed anoxically in potassium phosphate buffer (50 mM, pH 7.5) with 2 mM benzyl viologen. Staining was started with 5 mM acetaldehyde, 5 mM formate or pure hydrogen gas, respectively. Addition of dithionite reduced response time of the enzyme reaction. Results of peptide mass fingerprinting are displayed in S1 Table. The unmarked, second spot at the formate dehydrogenase staining developed before formate addition, probably due to hydrogen contamination of the gas phase. (TIF) [file pone.0115902.s003.tif]

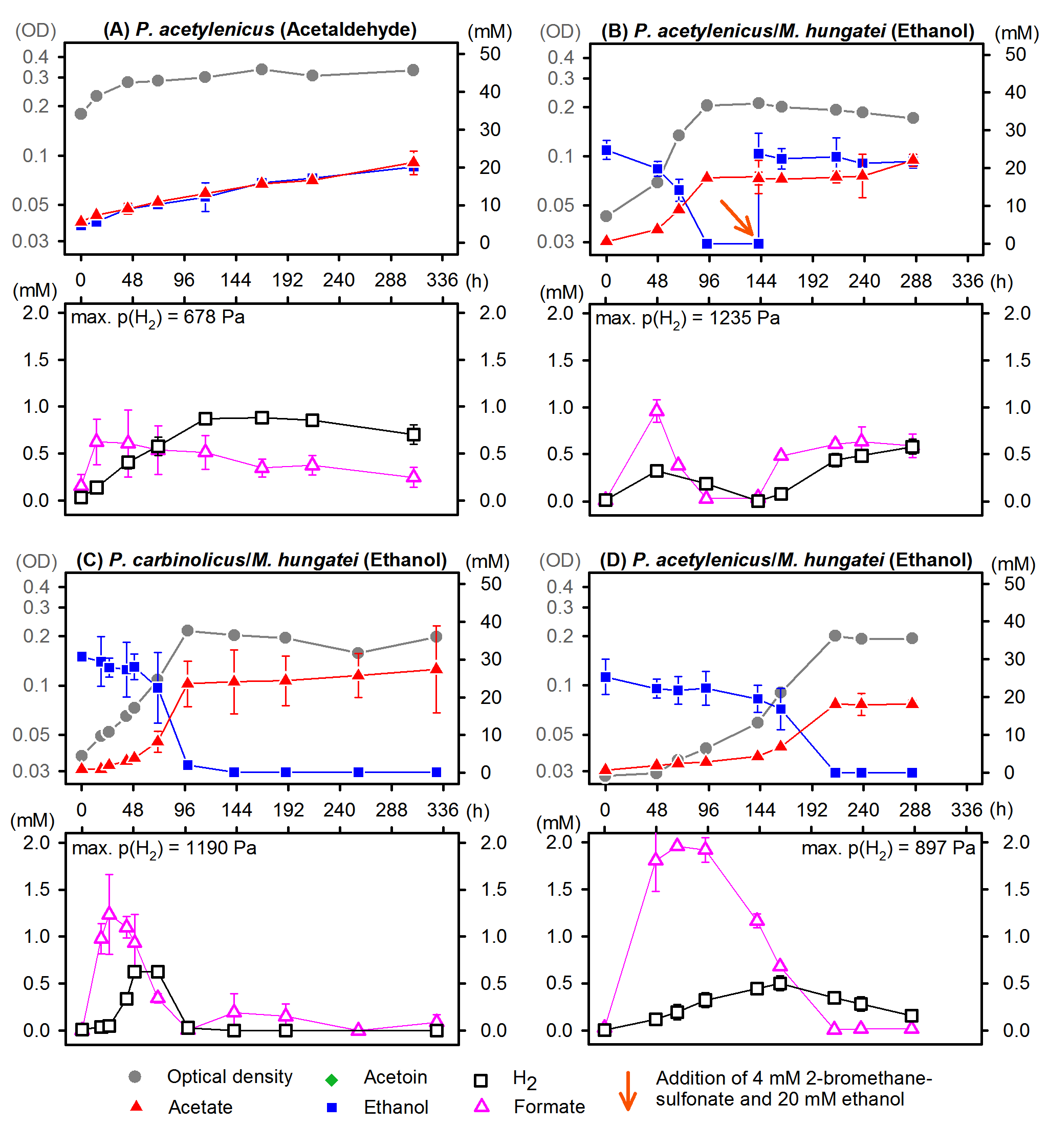

Supplement: S4 Fig — Growth of representative P. acetylenicus cultures degrading acetaldehyde (A) or ethanol (coculture with M. hungatei JF1; B,D) show same hydrogen or formate production and consumption pattern as the corresponding P. carbinolicus cultures (C, see Fig. 3 in main article). Inhibition of the methanogen by addition of 2-bromoethanesulfonate (BES, see arrow) and readdition of 20 mM ethanol as substrate (B) led to similiar levels of accumulated hydrogen and formate. (symbols: optical density (filled circles), ethanol (squares), acetate (triangles), hydrogen (open squares) and formate (open triangles)). (TIF) [file pone.0115902.s004.tif]

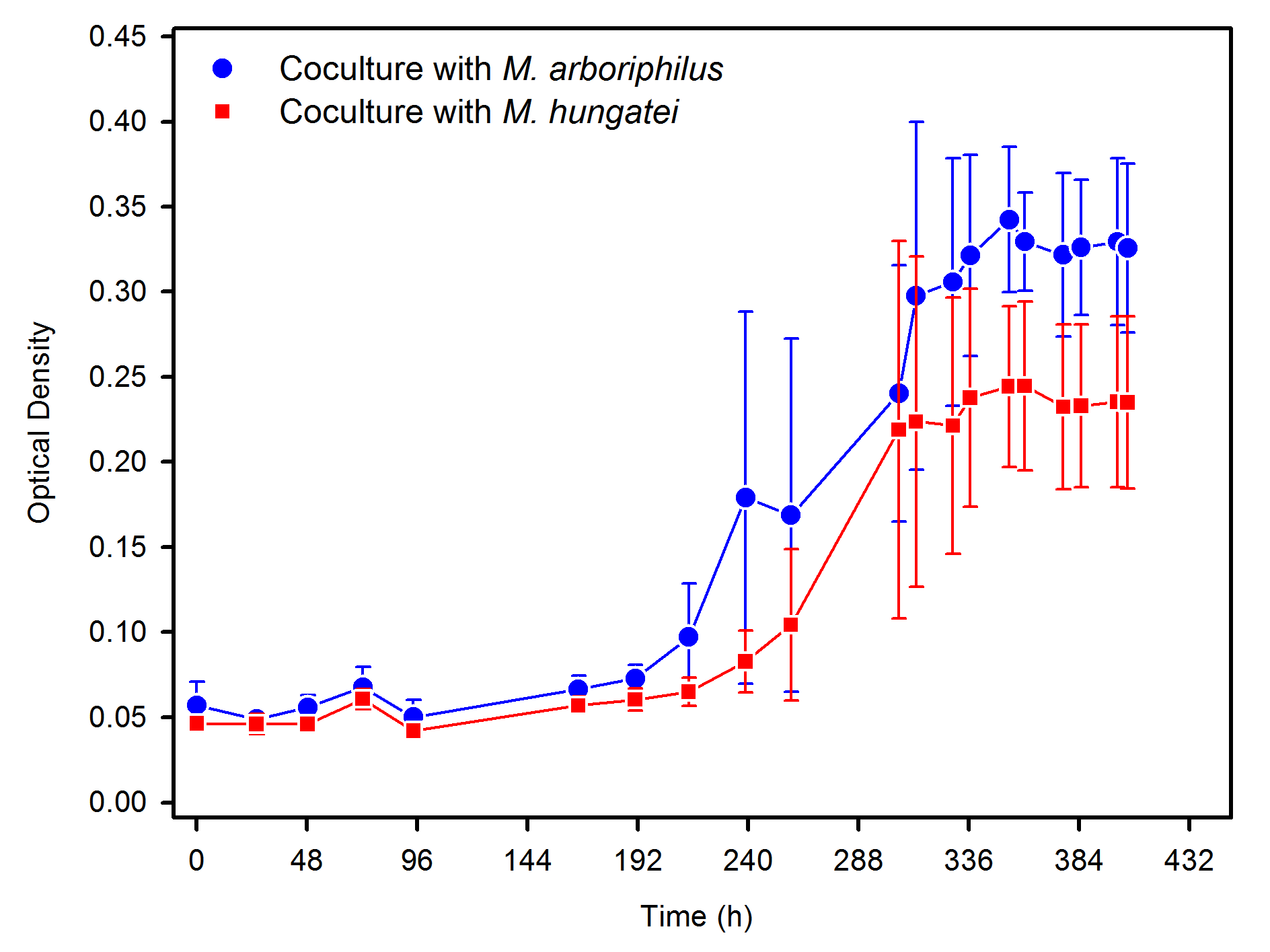

Supplement: S5 Fig — Growth of Pelobacter carbinolicus in coculture with different methanogenic partners on 20 mM ethanol: Methanobrevibacter arboriphilus (blue circles) and Methanospirillum hungatei M1h (red squares). Depicted are data obtained in triplicate cultures. (TIF) [file pone.0115902.s005.tif]
