# Supplementary material for: Degradation of Acetaldehyde and Its Precursors by Pelobacter carbinolicus and P. acetylenicus
Source: PLoS One. 2014 Dec 23;9(12):e115902. doi: 10.1371/journal.pone.0115902 (PMC4275255; doi:10.1371/journal.pone.0115902)
Supplement: S1 Table — Results of peptide mass fingerprinting to identify spots that were stained by non-acetylating acetaldehyde dehydrogenase (acetaldehyde: benzyl viologen oxidoreductase) (AADH), formate dehydrogenase (FDH) and hydrogenase (H2ase) activity as shown in S3 Fig . under different growth conditions. A score value above 50 is meant to be significant. (Abbreviations: Fd = ferredoxin, dep. = dependent, su = subunit). (DOCX) [file pone.0115902.s006.docx]

**Table S1.** Results of peptide mass fingerprinting to identify spots that were stained by acetaldehyde dehydrogenase (AADH), formate dehydrogenase (FDH) and hydrogenase (H_2_ase) activity as shown in Fig. S3 under different growth conditions. A score value above 50 is meant to be significant. (Abbreviations: Fd = ferredoxin, dep. = dependent, su = subunit)

| Protein spot | Staining activity | LocTag (Pcar_) | Putative Annotation | Score | Sequence coverage (%) |
| --- | --- | --- | --- | --- | --- |
| A1 | AADH (Mo) | 0377 | Pyruvate:Fd oxidoreductase | 172 | 18 |
|  |  | 0220 | aldehyde:Fd oxidoreductase | 79 | 10 |
|  |  | 1633 | NAD(P)-dep. hydrogenase (Fe-only) | 55 | 18 |
|  |  | 1636 | NADH dehydrogenase (su E) | 54 | 31 |
| A2 | AADH (Mo) | 0220 | Aldehyde:Fd oxidoreductase | 264 | 26 |
| A3 | AADH (Mo) | 1604 | NAD(P)-dep. hydrogenase (Fe-only) | 141 | 18 |
|  |  | 1633 | NAD(P)-dep. hydrogenase (Fe-only) | 140 | 23 |
| B1 | AADH (W) | 0665 | Aldehyde:Fd oxidoreductase (Mo-dep.) | 116 | 18 |
|  |  | 1604 | NAD(P)-dep. hydrogenase (Fe-only) | 113 | 29 |
|  |  | 0834 | NADH:ubiquinone oxidoreductase | 99 | 15 |
|  |  | 0456 | Aldehyde:Fd oxidoreductase (W-dep.) | 65 | 9 |
|  |  | 1633 | NAD(P)-dep. hydrogenase (Fe-only) | 59 | 22 |
|  |  | 0251 | Alcohol dehydrogenase | 58 | 8 |
| B2 | AADH (W) | 0665 | Aldehyde:Fd oxidoreductase (Mo-dep.) | 148 | 14 |
|  |  | 0251 | Alcohol dehydrogenase | 134 | 31 |
|  |  | 0456 | Aldehyde:Fd oxidoreductase (W-dep.) | 79 | 18 |
|  |  | 1634 | NADH dehydrogenase (su F) | 68 | 13 |
|  |  | 1633 | NAD(P)-dep. hydrogenase (Fe-only) | 49 | 6 |
| B3 | AADH (W) | 0665 | Aldehyde:Fd oxidoreductase (Mo-dep.) | 152 | 14 |
|  |  | 0255 | Alcohol dehydrogenase | 143 | 20 |
|  |  | 1594 | Alcohol dehydrogenase | 69 | 12 |
|  |  | 0835 | NAD-dep. formate dehydrogenase (α su) | 69 | 15 |
| C1 | H_2_ase (W) | 0665 | Aldehyde:Fd oxidoreductase (Mo-dep.) | 152 | 17 |
|  |  | 0835 | NAD-dep.formate dehydrogenase (α su) | 75 | 15 |
|  |  | 1594 | Alcohol dehydrogenase | 48 | 18 |
|  |  | 0255 | Alcohol dehydrogenase | 45 | 18 |
| D1 | H_2_ase (W) | 0220 | Aldehyde:Fd oxidoreductase | 152 | 20 |
|  |  | 1594 | Alcohol dehydrogenase | 60 | 11 |
|  |  | 1636 | NADH dehydrogenase (su E) | 43 | 31 |
| D2 | H_2_ase (W) | 1636 | NADH dehydrogenase (su E) | 116 | 40 |
|  |  | 0220 | Aldehyde:Fd oxidoreductase | 61 | 8 |
|  |  | 1846 | NADH:ubiquinone oxidoreductase | 43 | 13 |
| E1 | FDH (W) | 1604 | NAD(P)-dep. hydrogenase (Fe-only) | 123 | 8 |
|  |  | 1594 | Alcohol dehydrogenase | 80 | 9 |
|  |  | 1633 | NAD(P)-dep. hydrogenase (Fe-only) | 61 | 12 |
|  |  | 1605 | NAD(P)-dep. hydrogenase (Fe-only, cat. su) | 44 | 10 |
